# Investigation of Laser Macro- and Micro-Polishing on Fine-Grained Niobium Material for Superconducting Radio Frequency Cavities

**DOI:** 10.3390/ma18215034

**Published:** 2025-11-05

**Authors:** Florian Brockner, Laura Kreinest, Edgar Willenborg, Dirk Lützenkirchen-Hecht

**Affiliations:** 1 Fakultät 4-Physik, Bergische Universität Wuppertal, Gaußstr. 20, D-42119 Wuppertal, Germany; brockner@uni-wuppertal.de; 2 Fraunhofer Institut für Laser Technology ILT, D-52074 Aachen, Germanyedgar.willenborg@ilt.fraunhofer.de (E.W.)

**Keywords:** niobium, laser polishing, superconductivity

## Abstract

Fine-grained Nb metal sheets were successively laser macro- and micro-polished for a potential use of the so-prepared materials in superconducting radiofrequency cavities in particle accelerators. The laser-treated Nb surfaces were investigated by a combination of white light interferometry, optical profilometry, electron microscopy with X-ray spectroscopy, and X-ray diffraction to study the influence of the conditions during the laser treatments on the resulting surface topography, the crystallographic structure, and the chemical composition of the material samples. For optimum polishing conditions, smooth, wavy surfaces with a minimum surface roughness could be achieved. However, local defects such as carbon contamination, as well as holes and cracks in the surface, were found. For the different prepared surfaces, the maximum acceleration field gradients, i.e., the onset fields for field emission (E_On_), were determined, indicating that for smooth surface regions without defects, E_On_ may reach values of up to almost 1 GV/m, while for the pristine, rough surface and local defects such as particles and cracks, E_On_ is limited to values around 100 MV/m or less. The present study suggests that laser polishing should be considered as an alternative to conventional polishing strategies of niobium accelerator cavities.

## 1. Introduction

Almost all modern particle accelerators, such as the Large Hadron Collider at CERN [1], the European Spallation Source [2], or the European X-ray free-electron laser (X-FEL) [3], rely on superconducting radio-frequency (SRF) cavity resonators made of niobium. In order to enable the fabrication of high-performance cavities with sufficient accelerating field gradients of 30 MV/m or more, sophisticated processing techniques have been developed even for an application on an industrial scale. On a laboratory scale, values of about 50 MV/m, i.e., close to the theoretical limit [4], are feasible [5]. State-of-the-art manufacturing processes include chemical polishing to remove contaminations at the surface of the materials, and different heat treatments in high vacuum are used to reduce crystallographic defects and stress in the lattice (e.g., [5,6]). Elaborate polishing procedures, including buffered chemical etching in hydrofluoric acid and nitric acid [7], centrifugal barrier polishing, and electrochemical polishing, again employing HF [8], are used to transform the initially technically rough starting surfaces to a mirror-like finish, removing also the crystallographic defects induced by forging and mechanical polishing. These smooth surfaces are an intrinsic prerequisite to suppress parasitic electron field emission processes that may limit the applicable acceleration fields [9]. Last but not least, residues from the polishing solutions have to be removed from the cavity surfaces by additional cleaning steps subsequently, e.g., by high-pressure water rinsing [7,8] or dry ice cleaning [10].

It is important to note that current preparation routes for Nb cavities consume large volumes of toxic and harmful hydrofluoric acid for chemical polishing and electropolishing, and the heat treatments of the cavities are performed in ovens with wall-plug powers of several kW for many hours or even days. Those treatments are needed to remove hydrogen incorporated in the niobium material, which tends to form non-superconducting Nb-hydrides, and due to the contact with aqueous electrolytes, the formation of surface oxides is facilitated. In order to allow a more economic construction and operation of future accelerators, such as the planned Future Circular Collider [11] or the International Linear Collider [12], a further increase in the field strength and a reduction in the electric power dissipation are necessary, and, in accordance with the Paris Agreement [13], new, innovative, energy-efficient, and environmentally friendly preparation methods for the accelerators are needed. Facile, economic, and energy-efficient cavity processing techniques may also facilitate a more widespread use of accelerator technology, e.g., in industrial or medical applications.

In this context, dry laser polishing processes appear to be of potential interest: intense, pulsed, or continuous laser beams are used to melt the surfaces of the workpieces, effectively removing contaminations such as the above-mentioned hydrides and oxides and smoothing the machined surfaces [14,15,16]. Compared to the conventional cavity treatments, the efficient energy transfer to the near-surface region of the workpieces allows a substantial reduction in the applied energy, and any toxic or potentially harmful liquids are entirely avoided. However, the interactions of the laser beams with the materials of the workpiece are very complex and need to be optimized for each material individually [16,17,18,19,20]. However, laser polishing provides excellent opportunities for a tailored treatment; for example, energy regulation is easily possible by the variation in the laser power, the number of used laser pulses, repeating frequency, laser focus size, and the scan speed on the workpiece, so that the heat introduced to the material can be precisely controlled, and thus, any unwanted bulk heating of the material can be avoided. Surface cleaning processes are also possible employing laser treatments [21], for which commercial solutions are available [22]. In relation to laser treatments of cavity materials for accelerators, it was recently shown that scratches remaining after the conventional polishing processes can be removed purposefully, allowing for a substantial increase in accelerating field gradients and suppressing parasitic electron field emission [23,24].

Up to now, laser polishing processes have been restricted to laboratory-sized setups, as proof-of-principle studies were the focus of interest, and accordingly, the polished surface areas are of the order of several mm^2^ only [25,26]. It is thus of high interest whether the approach can be transferred to a near-technology scale, with a fast treatment of larger surface areas. In the present study, therefore, we have applied laser macro- and micro-polishing to an untreated, fine-grained Nb sheet directly taken from the fabrication of the Tesla cavities for the X-FEL and studied in detail the effects of the laser polishing treatments on the resulting surface structures on the Nb material and provide evidence that laser polishing might indeed be used for the large-scale processing of Nb cavities.

## 2. Materials and Methods

### 2.1. Materials

Pieces of ca. 21 × 21 mm^2^ were cut from a 3 mm thick fine-grain niobium sheet with 85.25 to 86.8 grains per mm^2^, taken from the series production of the TESLA cavities for the European XFEL by high-pressure waterjet cutting at DESY, Hamburg. The sheet with a surface roughness R_a_ = 0.97–1.43 μm was used for laser polishing without any mechanical or chemical pre-polishing in order to examine the feasibility of dry laser polishing treatments as a replacement for the conventional polishing strategies applied so far.

### 2.2. Laser Polishing

The laser polishing processes were all carried out employing the equipment available at the Fraunhofer ILT (Aachen, Germany) [27]. All treatments were performed under an inert Ar-gas atmosphere with oxygen and humidity levels well below 500 ppm. A galvanometric laser scanner (hurrySCAN30, ScanLab GmbH, Puchheim, Germany) was used to move the beam in a meandering pattern over the niobium surface to be polished, and a computer-controlled, 5-axis portal machine positions the workpieces in the beam. While a CW fiber laser with an infrared wavelength of 1075 nm (redPOWER, SPI Laser Ltd., Southhampton, UK) was used for the macro-polishing, a Q-switched disk laser (TruMicro 7050, Trumpf, Ditzingen, Germany) with a wavelength of 1030 nm and a maximum average laser power of 520 W was employed for subsequent micro-polishing steps. Both laser optics employed a circular shape with a flat-top intensity distribution. The focus of the two laser beams can be manipulated by a zoom telescope. All important polishing parameters, such as the laser power, the feed rate, the beam diameter, the track offset as well as the number of polishing cycles, were systematically varied during the experiments. More details of the laser polishing setups are given, e.g., in refs. [28,29,30,31]. For each polishing condition, a test field of 5 × 5 mm^2^ was treated and subsequently analyzed with white light interferometry, optical profilometry, electron microscopy with energy-dispersive X-ray analysis (SEM/EDX), and X-ray diffraction, respectively. Some selected samples were laser-polished on larger areas of up to 15 × 15 mm^2^. Up to now, in total, more than 50 different test fields have been prepared and analyzed.

### 2.3. Materials Analysis

The surface topography of the investigated niobium surfaces was investigated employing white light interferometry (WLI; Nexview NX2 (Zygo Corporation, Middlefield, CT, USA), with lateral and vertical resolution of about 3 μm and 1 nm, respectively. WLI data of 1 mm^2^ areas in the center of the laser-polished regions are analyzed with a profile filter so that the roughness of the sample is obtained as a function of the spatial wavelength, as described in more detail in ref. [28]. Furthermore, optical profilometry (OP) was used to further study the surfaces of the samples with an FRT Metrology MicroProf system (now Camtek, Bergisch Gladbach, Germany) with a lateral resolution of ca. 2 μm and a vertical resolution of ca. 3 nm. Scanning electron microscopy was carried out in a JEOL JSM-6510 system (JEOL JSM 6510, JEOL Ltd., Tokyo, Japan) equipped with a Thermo Fisher Scientific (Waltham, Massachusetts, USA) EDX system (Noran 7, silicon drift diode) with an energy resolution of about 120 eV, which allows detection of light elements. X-ray diffraction measurements were performed on a Malvern Panalytical (Almelo, the Netherlands) multipurpose diffractometer with Cu Kα radiation and a multi-strip detector in the Bragg–Brentano parafocusing geometry.

### 2.4. Parasitic Field Emission Studies

Field emission measurements of the pristine and the different laser-polished samples were performed in a dedicated measurement system, generally operated under ultrahigh vacuum [32]. The system has a fixed anode and a cathode holder with the Nb sample, which can be moved in a computer-controlled raster pattern. In the present study, truncated tungsten cones with a diameter of 150 μm were used to measure the onset fields for electron field emission and the resulting currents as a function of the position on the sample, locally and on average.

## 3. Results and Discussion

A pre-screening of the obtained surface topographies after laser polishing treatments was used to select an appropriate focus spot of the laser and a closely connected line spacing of the laser on the surface during laser polishing, both for macro- and micro-polishing. Variation in the laser spot size (D_L_) included the range from 250 μm to 500 μm, and line spacings (track offset d_y_) from 50 μm to 100 μm. Furthermore, the scan speed (line feed v_s_) was varied between 25 mm/s and 100 mm/s for the macro-polishing (MP). Furthermore, the number of polishing scans (*n*) was also varied, with the scan direction being rotated by 90 degrees after each scan. Best results were obtained for D_L_(MP) = 250 μm, d_y_(MP) = 50 μm, and v_s_(MP) = 25 mm/s, so these parameters were used for all subsequent laser macro-polishing treatments (see Supplemental Information, Figure S1). In particular, the largest investigated laser power and the lowest scan speed lead to a larger melt pool with a higher temperature, so that the applied heat may lead to a more pronounced smoothing of the treated surfaces.

**Figure 1:**
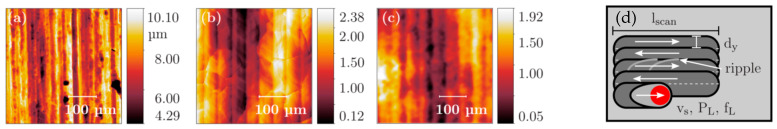
Topography of the niobium sheet investigated with white light interferometry prior to any polishing (**a**), after macro polishing (P_L_(MP) = 240 W, one horizontal (h, x-direction) + one vertical (v, y-direction) scan) (**b**), and after an additional micro-polishing (P_L_(μP) = 80 W, again two cycles in h and v) (**c**). (**d**) represents a scheme of how the rippled structure of the surface after laser polishing is generated in the x- and y-directions. The white arrows indicate the movements of the laser beam on the sample surface.

Similarly, the micro-polishing (μP) parameters were first screened, with resulting focus diameter of D_L_(μP) = 280 μm, d_y_(μP) = 30 μm, and v_s_(μP) = 1000 mm/s with a pulse repetition frequency of 20 kHz of 120 ns laser pulses. Some typical white light interferometry results are compiled in Figure 1, where the topography of the untreated niobium sheet (Figure 1a) is compared to that of a macro-polished area with a laser power of 240 W and a vertical and a second horizontal polishing scan (Figure 1b). Obviously, the roughness of the macro-polished sample is substantially reduced, i.e., the grooves from the mechanical processing of the samples can be efficiently removed by macro-polishing. However, although the surface contour appears much more homogeneous compared to the untreated sample, i.e., the polycrystalline nature of the sample becomes visible and a substantial reduction in surface roughness is obvious, the tracks remaining from the laser macro-polishing are clearly visible, despite the fact that two scans, one in a vertical and a second in a horizontal direction, have been performed (see, e.g., [29]). Those structures are a remainder of the localized melting of the material in the laser focal spot and its re-solidification, as schematically shown in Figure 1d. However, a further decrease in roughness and an increase in homogeneity can be noted after two additional micro-polishing treatments (Figure 1c).

**Figure 2:**
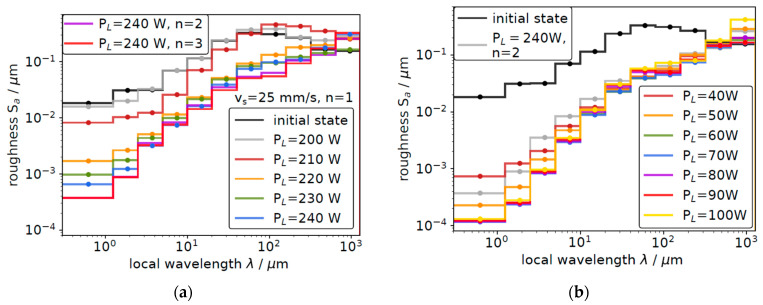
Evaluation of the average surface roughness on representative areas of 1 mm^2^ as a function of the lateral length scale as measured with light interferometry. (**a**) Roughness evaluation after macro-polishing for different laser powers, P_L,_ and different numbers of macro-polishing (*n* = 1, 2, 3) at a speed of 25 mm/s. (**b**) Roughness evolution after an additional micro-polishing treatment for different average laser powers and a processing speed of 1000 mm/s in comparison to the initial surface topography of the untreated sample and the macro-polished sample (P_L_(MP) = 240 W, *n* = 2).

The effects of multiple laser polishing treatments and the laser power are compiled in Figure 2. Here, the average roughness on different length scales as determined from a detailed analysis of the white light interferometry images is depicted for both the macro-polishing (Figure 2a) and the effect of an additional micro-polishing with a pulsed laser of different laser power (Figure 2b). As can be seen, a laser power of at least 210 W is required to induce a noticeable reduction in the roughness on a μm length scale after a single MP treatment (dark red curve in Figure 2a), and a further increase in the laser power to 240 W allows us to substantially reduce roughness on all length scales (blue curve in Figure 2a). If a second MP treatment is applied with P_L_(MP) = 240 W, the roughness may be further reduced, i.e., by rotating the meander pattern by 90 degrees, a second MP treatment may reduce the tracks from the first polishing cycle as well as the remaining roughness features from the pristine sample (pink curve in Figure 2a). A third and even more MP cycles do not substantially alter the surface topography, as can be deduced from the light red curve in Figure 2a, so that all laser MP treatments were performed with two MP cycles and a laser power of 240 W (see also Figure S1 in the Supplementary Material). In the context of cavity production and the objective to suppress any parasitic field emission from the laser-polished surfaces, in particular, the roughness on short length scales is a key issue, and this is effectively reduced by a laser power of 240 W. For slow scan speed and high laser power, however, roughness on larger length scales in the order of mm increases, indicating that in this particular case, too much heat is introduced (see also Figure S1 in the Supplementary Material).

For a subsequently executed micro-polishing treatment, again, a threshold of at least 50–60 W laser power is necessary to further modify the topography of the macro-polished Nb samples (see Figure 2b). As can be seen, an average laser power of more than 90 W leads to an increase in surface roughness for larger local wavelengths, most probably by inducing stress in the treated metal sheet, induced by rapid heating and cooling cycles [29]. Optimum results are obtained for a laser power of P_L_(μP) = 80 W and again, one vertical plus one horizontal meander scan.

**Figure 3:**
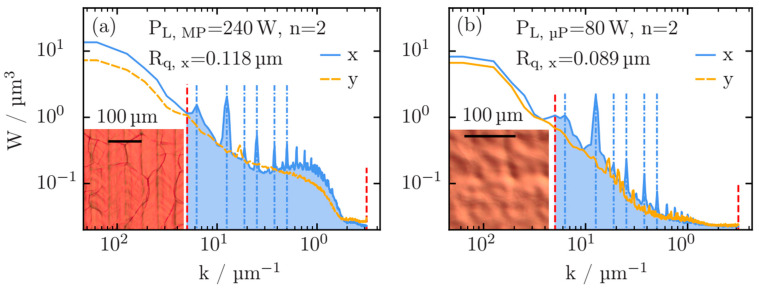
(**a**) Power spectral density functions (W) of an only macro-polished Nb sample (P_L_(MP) = 240 W, n = 2), and (**b**) a second sample subsequently micro-polished (P_L_(μP) = 80 W, *n* = 2). The x- and y-directions correspond to a direction perpendicular and parallel to the last laser polishing treatment, respectively, and are treated separately. In the inserts, results of an inspection with light microscopy are shown for the two samples. The dashed blue lines correspond to peaks in the respective PSDFs at λ = 13 μm, 17 μm, 25 μm, 33 μm, 50 μm, and 100 μm, and the red vertical lines present the k-range for which the average roughness values (R_q_) are calculated (λ_min_ = 2 μm, λ_max_ = 120 μm).

In order to investigate the roughness features of the laser-treated surfaces in more detail, optical profilometry was applied, and in Figure 3, the results of the OP data evaluation are presented. More specifically, the power spectral density functions (PSDF), a measure of the roughness depending on the lateral frequency (wave number k = 2π/λ) [33,34]. In general, the PSDF represents the Fourier transform of the autocorrelation function R(ξ) (Equation (1)),

(1) $$R(\xi )=\underset{L\to \infty }{\lim}\frac{1}{L}\int_{0}^{L}z\left(x\right)z\left(x+\xi \right)dx.$$

R(ξ) provides a measure of the probability of finding a point of identical height *z*(*x*) at a distance *x + ξ*, and the PSDF is given by

(2) $$W(k)=\frac{1}{2\pi }\int_{-\infty }^{\infty }R\left(\xi \right)\exp\left(-ik\xi \right)d\xi .$$

Thus, peaks in the PSDF correspond to periodic wave structures (wave number *k*) and are thus well-suited to identify traces of the tracks from the laser polishing as well as other laser-induced periodic structures on the surfaces [35]. Those periodic structures are not detectable by simply measuring the roughness as a function of their length scale, as depicted in Figure 2. Here we discriminate between the PSDF perpendicular to the direction of the last laser polishing scan (x-direction) and the PSDF parallel to the last laser polishing (y-direction).

In Figure 3a, the PSDF calculated after the macro-polishing (P_L_(MP) = 240 W, *n* = 2) is shown together with an optical inspection with a light microscope. While the PSDF is smooth along the polishing tracks and substantially decaying towards increasing wavenumber (decreasing length scale λ), distinct peaks are present in the x-direction. These peaks, located at λ = 13 μm, 17 μm, 25 μm, 33 μm, 50 μm, and 100 μm, are the remainder of the polishing tracks and can also be seen in the light microscopy image in the inset of Figure 3a. The largest amplitude in the PSDF at 50 μm directly corresponds to the used line spacing d_y_(MP) = 50 μm. For the direction along the polishing tracks (y-direction), any distinct peaks are absent, indicating that the melting and re-solidification of the niobium results in a reduced roughness of the laser-treated material already after the macro polishing.

After two additional micro-polishing treatments, the roughness further decreases in both the x- and y-directions; however, remnants of the frequency spectrum of the macro-polished sample still persist. Direct traces of the line spacing in the micro-polishing are expected for λ = d_y_(μP) = 30 μm. However, still, the peak corresponding to the line spacing of the macro-polishing procedure is dominating the PSDF in the x-direction. Smaller peaks are also visible in the y-direction, which are a fingerprint of the overall smooth but wavy surface topography of the macro- and micro-polished sample. Here, no indications for line traces are detectable by light microscopy (insert of Figure 3b). The roughness calculated by integration of the PSDF from λ_min_ = 2 μm to λ_max_ = 120 μm along the x-direction, i.e., with an enhanced roughness, leads to a value of 0.118 μm for the macro-polished sample (Figure 3a) and only 0.089 μm for the macro- and micro-polished sample (Figure 3b). The most obvious decrease in roughness is observed in the length scale range below about 30 μm, which is most crucial for the suppression of parasitic electron field emission and breakdown of superconductivity in niobium cavity materials. In order to further reduce lines and ripples, a rotation of the sample during laser treatment and more sophisticated laser tracks on the polished workpiece could be considered (see Figure 1). Furthermore, the intensity distribution of the laser beam could be adapted.

**Figure 4:**
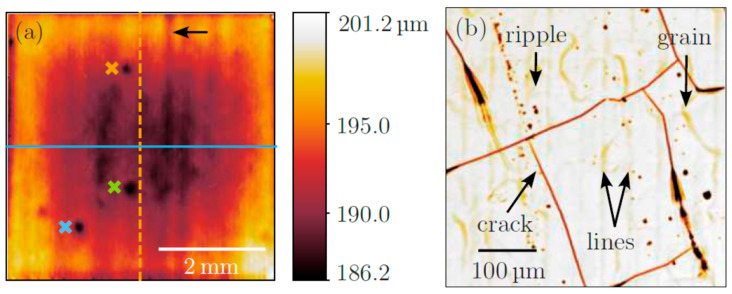
(**a**) White light interferometry of the entire surface of a laser macro- and micro-polished Nb sheet. Besides a wavy surface appearance, distinct defects are detectable, i.e., a remaining scratch (black arrow) and circular pits (colored crosses). Two line profiles (blue horizontal line and orange vertical line) are indicated, which were used to measure any macroscopic deformation induced by the laser polishing procedure. (**b**) Light microscopy of defects. Again, ripples as well as defects are detected and indicated accordingly. Traces of scan lines, as well as cracks in the surface and the grain structure of the material, are visible.

An example of a full-field white-light interferometry image of a 5 × 5 mm^2^ sample is shown in Figure 4a. Despite the fact that the entire surface was prepared with optimized laser polishing conditions, a large inhomogeneity can be stated. First of all, on a large scale, there are still some periodic features remaining from the macro-polishing, in the present case, in the up/down direction. Second, there are some remaining defects from the original niobium sheet, i.e., a vertical scratch from the rolling processes (black arrow in Figure 4a) and some pits marked by colored crosses. The depth of the pits was determined to be about 3.6 μm (green cross), 4.5 μm (orange cross), and 6.4 μm (blue cross), respectively. Obviously, laser polishing alone cannot remove all the different defects that are present on the pristine, original niobium sheet, and some mechanical or chemical pre-polishing appears to be required. For two line profiles, the average height variation was also determined, resulting in values of 8.7 μm (horizontal blue profile) and 7.7 μm (vertical orange profile), indicating that the sheet was slightly deformed or bent due to the laser polishing, or more material was evaporated from the center of the sample in the course of the laser processing.

In addition to the ripples, lines, and point defects, visible light microscopy proves the grain structure of the material, as well as the presence of cracks in the laser-treated material. The latter may appear because extremely high temperatures are needed for the melting of niobium, with a bulk melting temperature of 2477 °C (ref. [36], p. 51). Furthermore, for high-quality polishing results, the temperature should be as high as possible, i.e., close to the boiling point of Nb (4927 °C) [37]. Limited by heat dissipation to the environment of the sample, thereby the time for the existence of the melt is maximized, and polishing effects are optimized. However, heating and cooling occur on time scales of microseconds or less, so extreme heating and cooling rates result, and the thermal loads may induce mechanical stress in the material, finally causing cracks at the surfaces. Although these cracks are critical in view of the use of laser-polished samples in acceleration structures, crack formation may be avoided, e.g., by some moderate preheating of the laser-treated samples to slow down in particular the cooling processes, a further optimization of the laser processing, or an additional heat treatment after the laser polishing.

**Figure 5:**
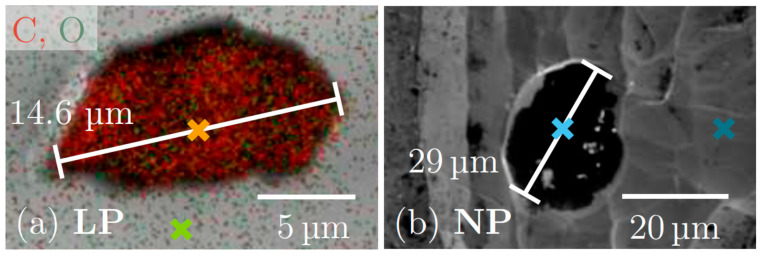
(**a**) SEM micrograph of a defect on the surface of a laser-polished Nb sample, with superimposed EDX signals of carbon (red dots) and oxygen (green dots). A spot analysis was performed for spots marked by orange and green crosses, yielding 48.0 ± 1.5 atomic % carbon and 30.8 ± 1.7 atomic % oxygen on the particle, and 16.3 ± 1.8 atomic % carbon and 9.0 ± 1.7 atomic % oxygen in the polished area. In addition, the remainder is niobium in both cases. (**b**) SEM micrograph of an untreated, pristine Nb sheet with a defect. Point analysis yields 27.9 ± 1.0 atomic % carbon and 15.4 ± 0.7 atomic % oxygen on the particle (bright blue cross), and 18.5 ± 1.1 atomic % carbon and 7.1 ± 0.5 atomic % oxygen in the pristine Nb surface area besides (dark blue cross).

Further point defects detected after laser polishing appear to be related to chemical contamination. Although the specification for the pristine material only allows 2 μg carbon/g niobium, electron-excited X-ray analysis proves substantial amounts of C on the surface of the pristine and the laser-treated Nb (see Figure S2 in the Supplementary Material). While the average concentration determined for oxygen slightly decreases after the laser polishing, indicating the effectiveness of the protecting noble gas atmosphere, the concentration of carbon substantially increases, reaching a value of more than 7 atomic %. This can be explained by the presence of carbon inclusions in the Nb material that have a smaller density than metallic niobium and thus float on the liquid metal during the laser melting and reside at the surface after re-solidification. Examples of such carbon contaminations in the laser-polished sample and in the pristine, untreated material are shown in Figure 5a,b. According to the SEM analysis, the size of both defects is on the order of 20 μm, and the particle detected after laser polishing is clearly mainly composed of carbon. A detailed analysis typically provides carbon concentrations of 30–40 at. %, and 15–30 at. % oxygen, while substantially lower concentrations are found on average. On a particle-free Nb surface, typically only about 10–15 at. % C and less than 10 at. % O were detected.

As can be seen in the SEM micrograph of the untreated, pristine Nb sheet, several smaller particles are present on the surface of the material, probably as residues from the rolling of the original sheet. The obtained results suggest that despite the high laser power applied in the consecutive macro- and micro-polishing steps, the LP does not appear to remove all residues on the Nb material, but at most redistributes them on the samples. Eventually, the laser processing needs to be carried out under vacuum, so that particulates may be evaporated and removed via the pumping units (see, e.g., [21,25]).

**Figure 6:**
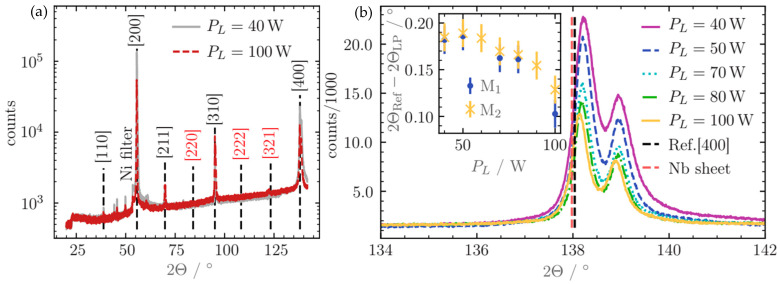
(**a**) XRD raw data for a micro-polished sample after two treatments with different laser powers. Bragg peaks are labeled with their Miller indices. Small diffraction features between 40° and 60° in the Bragg angle are due to a tungsten contamination of the Cu anode. Note the logarithmic scale of the diffractogram. (**b**) Detailed view of the (400) reflection for different micro-polishing conditions as indicated by different colored lines. In the inset, the (400) peak position is evaluated as a function of the laser power during micro-polishing for two samples, M1 and M2, with high reproducibility.

In order to investigate the crystallographic structure of the laser-polished Nb materials after the different treatments, X-ray diffraction experiments have been performed, and typical results are compiled in Figure 6. For the used X-ray wavelength of 0.154 nm (Cu Kα radiation), the penetration depth of the X-rays amounts to ca. 20 μm, so that only the near-surface region of the laser-polished samples is probed. It is important to keep in mind that the superconducting currents in a real Nb cavity only occur in a certain, even smaller depth, so that the diffraction measurements provide information about a practically relevant region of the laser-treated samples. The important parameter is the London penetration depth, which has a value of ca. 30–40 nm for niobium [38], i.e., substantially smaller compared to the depth probed by XRD. As can be seen in Figure 6a, all Bragg peaks expected for a polycrystalline body-centered cubic (bcc, space group 229) Nb sample with a lattice parameter a = 3.30 Å (ref. [36], p. 20) can be clearly detected, suggesting that the polycrystalline bcc structure persists after re-solidification. Looking in more detail at the positions of the Bragg peaks, smaller shifts are detectable, most prominently at larger Bragg angles. We thus analyzed the (400) reflection in more detail; see Figure 6b. As can be seen, the (400) peak is generally shifted towards larger Bragg angles after the different laser-polishing treatments, while the peak position of the untreated pristine Nb metal sheet corresponds well to the bcc-structure data of Nb (dashed vertical lines in Figure 6b). One may argue that the observed Bragg angle shifts arise from a possible misalignment of the diffraction geometry due to a bending of the investigated samples. However, such a bending would affect all Bragg peaks in a similar manner, which, on the other hand, is not observed.

In contrast, the observed peaks systematically shift to the theoretical position with increasing power of the micro-polishing treatment, suggesting that a compressive stress is preserved in the macro-polished Nb sheet. Such stress may result from the fast re-solidification processes of the metal in the cooling phase directly after the laser treatment. The XRD results suggest that this stress is reduced by the successive micro-polishing, where, in particular, the near-surface region of the laser-treated metal is remelted again. As can be deduced from the inset of Figure 6b, the Bragg peak shifts, and accordingly, the stress in the micro-polished samples decreases with increasing laser power during the micro-polishing treatment, again suggesting a laser power around 80–100 W as optimal.

**Figure 7:**
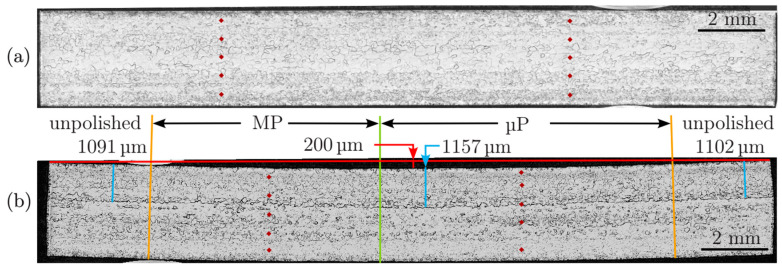
Light microscopy from a cross-section of the laser-polished niobium material (**a**) pristine Nb sheet and (**b**) laser-polished Nb sheet. Local hardness measurements were performed at the positions of the red dots, without any meaningful changes. Two polishing regions are displayed in (**b**)—one that is only macro-polished (left) and a second with macro- and micro-polishing treatments (right).

Finally, light microscopy experiments were performed on cross-sectioned samples in the pristine state and after different laser-polishing treatments on 15 × 15 mm^2^ areas. For this purpose, a laser-polished sample was bisected, with the intersection in a region with both only macro- and macro- and micro-polished areas and untreated regions. Before microscopic inspection, the cross-sections were etched by buffered chemical etching for 5–10 min to allow a more detailed visualization with an Olympus MX40 light microscope. Some representative results are presented in Figure 7. It should be noted that the laser-polished side is pointing downwards. First of all, it is obvious that due to the stress induced by the macro-polishing treatment, the entire niobium metal sheet is slightly deformed, as indicated by the curvature of the metal in the region of the laser treatments, as visible in Figure 7b, with a maximum deviation of ca. 0.2 mm on a length of 21 mm. Since the deformation is also found in a region that is only macro-polished, the deformation can be clearly assigned to the large heat introduced in the macro-polishing step. This deformation is in qualitative agreement with the peak shifts detected in the XRD measurements, although the latter are related to the microstructure of the materials.

Measurements of the hardness were performed using Zwick ZHV 30 zwickiLine HD (https://www.zwickroell.com/, accessed on 15 october 2025) at the positions marked by red points; however, there were no meaningful changes between the untreated metal and a laser polished region. More obviously, the boundary between the remelted regions and the pristine metal can be recognized by a change in the texture in the respective regions, at a depth of about 1100 μm, as indicated by the white bars in Figure 7b. Changes are visible in the textures of the respective regions, and the most intriguing feature is a larger average crystallite size of the laser-treated regions compared to the pristine Nb sheet. Thus, the laser treatment improves the crystallinity of the metal, reducing contributions of grain boundaries to superconductivity, and thus may improve the superconducting properties accordingly.

**Figure 8:**
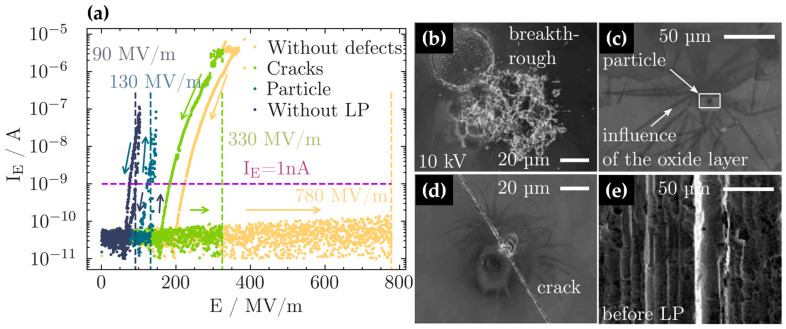
(**a**) Field emission measurements, i.e., the detected electron currents as a function of the applied electric field E in up- and downward directions as indicated by colored arrows, for different positions on the samples as indicated. The onset field was defined as the electric field for which an electron emission current of 1 nA is measured and is indicated on the respective curves. (**b**–**e**) SEM micrographs related to the different positions of field emission measurements are as follows: (**b**) sample position after current breakthrough at 780 MV/m (yellow curve in (**a**)), (**c**) sample position with particle on the surface (light blue curve in (**a**)), (**d**) sample position with a local crack (green curve in (**a**)), and (**e**) pristine sample (dark blue curve in (**a**)).

In order to verify the positive influence of the laser-polishing treatments on the parasitic field emission and the achievable acceleration fields, field emission measurements of some selected samples have been performed, i.e., the emission current was measured locally as a function of the applied electric field E; see Figure 8a. In general, as already suggested by previous experiments [23,24], a substantially larger onset field E_On_ for field emission was detected for the laser-polished surfaces in comparison to the untreated Nb surface. As can be seen in Figure 8a, the pristine Nb shows an onset field for field emission of about E_On_ = 90 ± 10 MV/m, at which a field emission current of 1 nA was detected. In contrast, on defect-free laser-polished surface regions, an acceleration field of almost E_On_ = 800 MV/m can be applied without any indications of electron field emission, suggesting that there are no protrusions, defects, or local inhomogeneities that may facilitate electron emission (see, e.g., ref. [39]). However, without current limitation, electron field emission may be activated by large accelerating gradients, resulting in a current breakthrough with currents in the range of several μA (explosive electron emission) and related morphological changes in the inspected surfaces, as can be seen in Figure 8b. Due to the strong local electron current, the surface is heated and Nb material is emitted, leading to an irreversible deterioration of the formerly smooth surface.

For the different defect structures discussed above, substantially lower onset fields for electron field emission were detected in general: particles (Figure 5, see the SEM micrograph in Figure 8c) lead to reduced onset fields of about E_On_ = 130 ± 20 MV/m; cracks (Figure 4b; see the SEM micrograph in Figure 8d) may allow electric fields of up to E_On_ = 330 ± 20 MV/m prior to field emission, while the pristine Nb surface with grooves and sharp surface features reveals an onset field of about E_On_ = 90 ± 10 MV/m, as already mentioned (see Figure 8e). In general, a hysteresis is observed in the current-voltage characteristics, i.e., if the electric field is reversed and scanned downwards, larger emission currents are detected (Figure 8a), which is a typical observation for activation processes triggered by the field emission. In light of the envisaged use of laser polishing treatment for a potential application to Nb cavity materials for applications in accelerators, where the accelerating field gradient should be as high as possible, it is important to note that the observed defect structures that reduce the onset field may be avoided, i.e., by improved cleaning procedures such as dry ice cleaning [10] and a second, localized laser polishing treatment [23,24], so that, thus, the use of laser polishing procedures appears very promising to suppress parasitic electron field emission processes.

## 4. Conclusions

Fine-grained Nb metal sheets were successfully laser-polished, employing successive laser macro- and micro-polishing steps, and investigated by a combination of different interferometric and microscopic techniques, as well as X-ray diffraction and field emission measurements. Under process conditions close to an industrial scale, i.e., using fast-scanning laser beams in an inert noble gas atmosphere protecting the delicate Nb metal surface from oxidation, smooth surfaces with a wavy character may be prepared under optimized laser polishing conditions. These smooth surfaces allow the application of acceleration field gradients of up to almost 800 MV/m prior to the onset of parasitic electron field emission, in contrast to only 90 MV/m for the untreated niobium and 100–300 MV/m on defect-containing areas of the laser-polished samples. Furthermore, the laser-polished surfaces also reveal an improved crystallographic structure with larger grains, and even after treatment in a technical environment, no substantial oxidation.

Defects that are still detectable on the laser-polished Nb surfaces, i.e., carbon contaminations and crack formation, need further investigation; however, e.g., heat treatments as well as additional laser-polishing procedures on preheated Nb may help to reduce or even prevent these defects. The presented results are encouraging to consider laser polishing as a promising alternative to conventional polishing treatments, as huge amounts of toxic and potentially harmful acids are avoided by entirely dry laser treatments. Furthermore, laser polishing efficiently uses the applied laser energy to modify the surface and volume microstructure of the treated metal, as the laser light is efficiently absorbed by the workpieces, so that an environmentally friendly and economic processing appears feasible. Superconducting properties of laser-polished fine-grain niobium samples have not yet been measured, but are, however, foreseen for the near future, to stress that such a strategy can be promising not only for future large-scale accelerator facilities, but also for small-scale accelerators, e.g., for industrial or medical purposes.

## Data Availability

The original contributions presented in this study are included in the article/Supplementary Material. Further inquiries can be directed to the corresponding author.

## Supplementary Materials

The following supporting information can be downloaded at: https://www.mdpi.com/article/10.3390/ma18215034/s1. Figure S1: Pre-screening of the parameters for the laser polishing treatments. (a) Macro polishing with a CW laser power of P_L_(MP) = 240 W, with different line feed rates (polishing speed v_s_(MP)). (b) Influence of the number of MP cycles with P_L_(MP) = 240 W on the resulting roughness of the Nb sample. (c) Influence of the laser power during micro-polishing on the roughness; Figure S2: Electron-excited X-ray analysis of a laser-polished niobium sheet. Substantial amounts of carbon and oxygen are detected if an area of about 1.2 mm^2^ is scanned. The average concentrations of niobium, carbon, and oxygen are 86.0 ± 0.4 atomic %, 4.1 ± 0.4 atomic %, and 9.9 ± 0.6 atomic % for the pristine sample, and 83.7 ± 0.4 atomic %, 7.2 ± 0.4 atomic %, and 9.1 ± 0.5 atomic % after the laser polishing. In the inset, a carbon-containing defect is shown, together with the overlaid signals of carbon (red dots) and oxygen (green dots).
