# Supplementary material for: Investigation of Laser Macro- and Micro-Polishing on Fine-Grained Niobium Material for Superconducting Radio Frequency Cavities
Source: Materials (Basel). 2025 Nov 5;18(21):5034. doi: 10.3390/ma18215034 (PMC12608439; doi:10.3390/ma18215034)
Supplement: Supplementary file 1 [file materials-18-05034-s001.zip › materials-3903220-supplementary.pdf]

materials

Article

Investigation of Laser Macro- and Micro-polishing on Fine-Grained Niobium Material for Superconducting Radio Frequency Cavities

Florian Brockner [1], Laura Kreinest [2], Edgar Willenborg[2], and Dirk Lützenkirchen-Hecht [1,*]

[1] Bergische Universität Wuppertal, Fakultät 4 - Physik, Gaußstr. 20, D-42119 Wuppertal, Germany; brockner@uni-wuppertal.de
[2] Fraunhofer Institut für Laser Technology ILT, D-52074 Aachen, Germany; laura.kreinest@heraeus.com (L.K.); edgar.willenborg@ilt.fraunhofer.de (E.W.)
* Correspondence: dirklh@uni-wuppertal.de

Supplemental Information

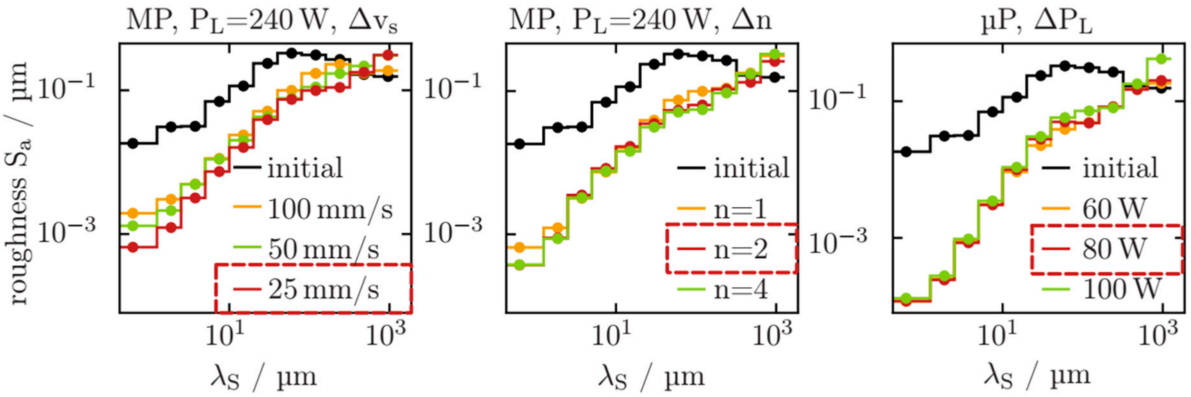

Figure S1. Pre-screening of the parameters for the laser polishing treatments. **(a)** Macro polishing with a CW laser power of P_L(MP) = 240 W, with different line feed rates (polishing speed v_s(MP)). **(b)** Influence of the number of MP cycles with P_L(MP) = 240 W on the resulting roughness of the Nb sample. **(c)** Influence of the laser power during micro-polishing on the roughness.

 https://doi.org/10.3390/ma18215034

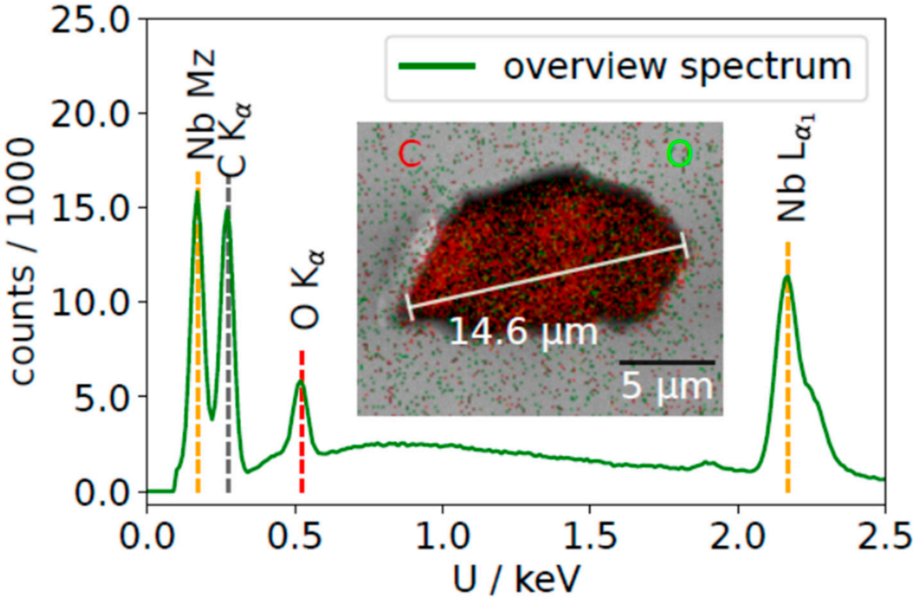

Figure S2. Electron-excited X-ray analysis of a laser-polished niobium sheet. Substantial amounts of carbon and oxygen are detected if an area of about 1.2 mm^2 is scanned. The average concentrations of niobium, carbon, and oxygen are 86.0 ± 0.4 atomic %, 4.1 ± 0.4 atomic %, and 9.9 ± 0.6 atomic % for the pristine sample, and 83.7 ± 0.4 atomic %, 7.2 ± 0.4 atomic %, and 9.1 ± 0.5 atomic % after the laser polishing. In the inset, a carbon-containing defect is shown, together with the overlaid signals of carbon (red dots) and oxygen (green dots).
